# Supplementary material for: Differential Proteomic Analysis of Listeria monocytogenes during High-Pressure Processing
Source: Biology (Basel). 2022 Jul 31;11(8):1152. doi: 10.3390/biology11081152 (PMC9405252; doi:10.3390/biology11081152)
Supplement: Supplementary file 1 [file biology-11-01152-s001.zip › biology-1771215-supplementary.pdf]

Supplementary Table S1. Bactericidal efficacy of *L. monocytogenes* exposed to HPP

Method	Pressure (MPa)	Time (s)	Surviving cell number (log CFU/mL)
HPP[1]	100	180	7.35 ± 0.07
	200	180	7.03 ± 0.18
	300	180	5.51 ± 0.08
	400	180	ND*

[1]HPP: High pressure processing

*ND: Means no detectable survivors by a direct plating procedure.

Values are mean ± S.D. of three repeated measurements

Supplementary Table S2. The number of proteins in each cluster by k-means.

Clusters	9G	9GE	3G	3GE
1 (HUR)*[1]	16 (2.812)*[2]	16 (0.996)	16 (2.865)	15 (1.027)
2 (UR)	32 (1.285)	32 (0.451)	32 (1.265)	33 (0.455)
3 (DR)	28 (-1.395)	28 (-0.483)	28 (-1.380)	28 (-0.479)
4 (HDR)	3 (-2.767)	3 (-1.104)	3 (-2.738)	3 (-1.097)

*[1]HUR: higher levels of up-regulated DEPs; UR: up-regulated DEPs; DR: down-regulated DEPs; HDR: higher levels of down-regulated DEPs

*[2]Clustering based on minimum distance in the parentheses of DEPs to centroid k^*.

Supplementary Table S3. Characteristics of DEPs grouping under k-means clustering algorithm

Protein IDs	Name (recommend name)	Entry name	Gene	Locus	‹H2/C›	‹H3/C›	‹H4/C›	9G	9GE	3G	3GE
					$\log_2$ (fold change)						
P0A3L1	Translation initiation factor IF-3	IF3_LISMO	*infC*	lmo1785	3.74811	3.932587	4.080817	1	1	1	1
P0DJP1	30S ribosomal protein S21	RS21_LISMO	*rpsU*	lmo1469	3.405787	3.558663	4.52361	1	1	1	1
P66125	50S ribosomal protein L27	RL27_LISMO	*rpmA*	lmo1540	2.980007	3.003287	3.31338	1	1	1	1
P66207	50S ribosomal protein L32-2	RL322_LISMO	*rpmF2*	lmo2047	2.931473	2.797813	3.29926	1	1	1	1
P66372	30S ribosomal protein S12	RS12_LISMO	*rpsL*	lmo2656	2.183107	3.04766	3.46765	1	1	1	1
P66383	30S ribosomal protein S13	RS13_LISMO	*rpsM*	lmo2608	2.815453	3.27682	3.895957	1	1	1	1
P66484	30S ribosomal protein S19	RS19_LISMO	*rpsS*	lmo2628	2.008937	2.318657	2.767097	1	1	1	1
P66503	30S ribosomal protein S20	RS20_LISMO	*rpsT*	lmo1480	2.341947	3.192303	3.417833	1	1	1	1
P66611	30S ribosomal protein S7	RS7_LISMO	*rpsG*	lmo2655	2.131183	2.154	2.81247	1	1	1	1
Q8Y443	50S ribosomal protein L24	RL24_LISMO	*rplX*	lmo2621	3.364153	3.64505	4.07866	1	1	1	1
Q8Y444	50S ribosomal protein L6	RL6_LISMO	*rplF*	lmo2617	2.023787	2.106403	2.223313	1	1	1	1
Q8Y459	30S ribosomal protein S9	RS9_LISMO	*rpsI*	lmo2596	2.649863	2.322057	2.593857	1	1	1	1
Q8Y699	30S ribosomal protein S16	RS16_LISMO	*rpsP*	lmo1797	2.235633	2.572573	2.816383	1	1	1	1
Q8YAR2	50S ribosomal protein L9	RL9_LISMO	*rplI*	lmo0053	2.45865	2.45913	2.53295	1	1	1	1
Q927M5	50S ribosomal protein L30	RL30_LISMO	*rpmD*	lmo2614	1.970263	2.87198	2.882087	1	1	1	1
Q8Y447	50S ribosomal protein L15	RL15_LISMO	*rplO*	lmo2613	1.891123	2.020003	2.393037	1	1	1	2
O53083	50S ribosomal protein L19	RL19_LISMO	*rplS*	lmo1787	0.902877	0.911553	1.019923	2	2	2	2
P0A485	50S ribosomal protein L31 type B	RL31B_LISMO	*rpmE2*	lmo2548	1.31141	1.073053	1.253447	2	2	2	2
P0DJM1	Chaperone protein DnaJ	DNAJ_LISMO	*dnaJ*	lmo1472	1.29662	1.561527	1.655297	2	2	2	2

P60426	50S ribosomal protein L2	RL2_LISMO	*rplB*	lmo2629	1.408533	1.407753	1.834237	2	2	2	2
P66054	50S ribosomal protein L11	RL11_LISMO	*rplK*	lmo0248	1.066717	1.361313	1.613543	2	2	2	2
P66103	50S ribosomal protein L20	RL20_LISMO	*rplT*	lmo1783	1.002737	1.567543	1.841793	2	2	2	2
P66330	30S ribosomal protein S10	RS10_LISMO	*rpsJ*	lmo2633	1.846163	1.74941	1.927417	2	2	2	2
P66352	30S ribosomal protein S11	RS11_LISMO	*rpsK*	lmo2607	1.082417	0.80424	1.047503	2	2	2	2
Q8Y3T8	Probable transaldolase 1	TAL1_LISMO	*tal1*	lmo2743	0.558987	1.671707	2.64027	2	2	2	2
Q8Y3X6	Nucleoid-associated protein lmo2703	Y2703_LISMO	*lmo2703*	lmo2703	1.20322	0.051417	1.990723	2	2	2	2
Q8Y440	50S ribosomal protein L3	RL3_LISMO	*rplC*	lmo2632	0.72686	0.763307	1.003223	2	2	2	2
Q8Y445	50S ribosomal protein L18	RL18_LISMO	*rplR*	lmo2616	1.134993	1.518597	2.099423	2	2	2	2
Q8Y449	Adenylate kinase	KAD_LISMO	*adk*	lmo2611	1.025257	1.484057	1.536273	2	2	2	2
Q8Y450	50S ribosomal protein L17	RL17_LISMO	*rplQ*	lmo2605	1.561887	1.624447	1.72547	2	2	2	2
Q8Y458	50S ribosomal protein L13	RL13_LISMO	*rplM*	lmo2597	1.405037	1.144157	1.278573	2	2	2	2
Q8Y4I3	Triosephosphate isomerase 1	TPIS1_LISMO	*tpiA1*	lmo2457	0.719637	1.06447	1.02544	2	2	2	2
Q8Y5G2	Pyridoxal 5'-phosphate synthase subunit PdxS	PDXS_LISMO	*pdxS*	lmo2101	0.972153	1.03581	1.146003	2	2	2	2
Q8Y5N9	Diaminopimelate epimerase	DAPF_LISMO	*dapF*	lmo2018	1.7819	1.277153	-0.41157	2	2	2	2
Q8Y673	DNA-directed RNA polymerase subunit omega	RPOZ_LISMO	*rpoZ*	lmo1826	0.529357	1.07498	1.128873	2	2	2	2
Q8Y6Y9	50S ribosomal protein L21	RL21_LISMO	*rplU*	lmo1542	1.310813	1.42591	1.375987	2	2	2	2
Q8Y7G7	Ribosome-recycling factor	RRF_LISMO	*frr*	lmo1314	0.965047	1.055713	1.142307	2	2	2	2
Q8Y7I9	S-ribosylhomocysteine lyase	LUXS_LISMO	*luxS*	lmo1288	1.933017	1.993717	1.97864	2	2	2	2

Q8Y7Q2	Phenylalanine--tRNA ligase alpha subunit	SYFA_LISMO	*pheS*	lmo1221	1.04695	1.01458	0.953827	2	2	2	2
Q8Y832	Ribitol-5-phosphate cytidylyltransferase	TARI_LISMO	*tarI*	lmo1086	0.93667	1.01323	1.291673	2	2	2	2
Q8Y8N0	ATP-dependent RNA helicase CshA	CSHA_LISMO	*cshA*	lmo0866	1.66747	1.576033	1.561057	2	2	2	2
Q8YAD5	Putative septation protein SpoVG 1	SP5G1_LISMO	*spoVG1*	lmo0196	0.13688	1.118607	1.292003	2	2	2	2
Q927L2	50S ribosomal protein L22	RL22_LISMO	*rplV*	lmo2627	1.449187	1.123487	1.33146	2	2	2	2
Q927L4	50S ribosomal protein L16	RL16_LISMO	*rplP*	lmo2625	1.408897	1.252623	1.750123	2	2	2	2
Q927L7	50S ribosomal protein L14	RL14_LISMO	*rplN*	lmo2622	1.274777	1.037983	1.08422	2	2	2	2
Q92C24	30S ribosomal protein S15	RS15_LISMO	*rpsO*	lmo1330	1.119627	1.131923	1.077357	2	2	2	2
Q9RGX0	Global transcriptional regulator Spx	SPX_LISMO	*spx*	lmo2191	1.016457	1.18597	1.7273	2	2	2	2
Q9RQI6	ATP-dependent Clp protease proteolytic subunit	CLPP_LISMO	*clpP*	lmo2468	1.04442	1.151423	1.41005	2	2	2	2
P0A355	Cold shock-like protein CspLA	CSPA_LISMO	*cspLA*	lmo1364	-0.6988	-0.94461	-1.24291	3	3	3	3
P0A357	Cold shock-like protein CspLB	CSPB_LISMO	*cspLB*	lmo2016	-2.02987	-2.77131	-3.23491	3	3	3	3
P0A4Q8	UPF0145 protein lmo0208	Y208_LISMO	*lmo0208*	lmo0208	-1.56063	-0.56337	-1.96188	3	3	3	3
P0DJL8	Alanine racemase	ALR_LISMO	*alr*	lmo0886	-0.94874	-0.85235	-1.03272	3	3	3	3
P58817	Aspartyl/glutamyl-tRNA(Asn/Gln) amidotransferase subunit C	GATC_LISMO	*gatC*	lmo1756	-2.3375	-1.93009	-2.18179	3	3	3	3
P64032	Elongation factor P	EFP_LISMO	*efp*	lmo1355	-1.67208	-1.82306	-1.96196	3	3	3	3
Q8Y454	Energy-coupling factor transporter ATP-binding protein EcfA1	ECFA1_LISMO	*ecfA1*	lmo2601	-2.1803	-2.61206	-3.00254	3	3	3	3
Q8Y4A6	Homoserine kinase	KHSE_LISMO	*thrB*	lmo2545	-1.24487	-0.96059	-2.04339	3	3	3	3

Q8Y4C2	ATP synthase epsilon chain	ATPE_LISMO	*atpC*	lmo2528	-0.84242	-1.13519	-1.25918	3	3	3	3
Q8Y4L2	Glycine cleavage system H protein	GCSH_LISMO	*gcvH*	lmo2425	-0.82362	-1.45353	-1.5876	3	3	3	3
Q8Y4L8	Methionine import ATP-binding protein MetN 2	METN2_LISMO	*metN2*	lmo2419	-0.84189	-0.83028	-1.07181	3	3	3	3
Q8Y564	Uroporphyrinogen decarboxylase	DCUP_LISMO	*hemE*	lmo2212	-1.05871	-1.12868	-1.20248	3	3	3	3
Q8Y5L9	UDP-N-acetylmuramoyl-L-alanyl-D-glutamate--2,6-diaminopimelate ligase	MURE_LISMO	*murE*	lmo2038	-0.68787	-1.08645	-1.25035	3	3	3	3
Q8Y5R1	Deoxyribose-phosphate aldolase	DEOC_LISMO	*deoC*	lmo1995	-1.42714	-1.38182	-1.53731	3	3	3	3
Q8Y5S8	Ribonuclease Z	RNZ_LISMO	*rnz*	lmo1977	-1.05298	-0.34887	-0.96692	3	3	3	3
Q8Y6A2	Ribosome maturation factor RimM	RIMM_LISMO	*rimM*	lmo1793	-0.70073	-1.103	-1.08827	3	3	3	3
Q8Y6V4	UPF0173 metal-dependent hydrolase lmo1577	Y1577_LISMO	*lmo1577*	lmo1577	-0.6953	-0.98357	-1.26489	3	3	3	3
Q8Y6V9	Acetyl-coenzyme A carboxylase carboxyl transferase subunit alpha	ACCA_LISMO	*accA*	lmo1572	-0.84724	-1.04496	-1.14232	3	3	3	3
Q8Y6W7	Formamidopyrimidine-DNA glycosylase	FPG_LISMO	*mutM*	lmo1564	-1.14561	-1.12913	-1.49144	3	3	3	3
Q8Y6X2	Threonine--tRNA ligase	SYT_LISMO	*thrS*	lmo1559	-0.64366	-0.91749	-1.08048	3	3	3	3
Q8Y6X4	Glutamyl-tRNA reductase	HEM1_LISMO	*hemA*	lmo1557	-1.99937	-2.12011	-2.11109	3	3	3	3
Q8Y6Z9	S-adenosylmethionine:tRNA ribosyltransferase-isomerase	QUEA_LISMO	*queA*	lmo1531	-0.95088	-0.89654	-1.10313	3	3	3	3
Q8Y7C6	Transcription antitermination protein NusB	NUSB_LISMO	*nusB*	lmo1359	-1.14637	-1.23831	-1.30993	3	3	3	3
Q8Y7H7	LexA repressor	LEXA_LISMO	*lexA*	lmo1302	-1.08714	-1.32471	-1.36438	3	3	3	3
Q8Y7P0	UvrABC system protein C	UVRC_LISMO	*uvrC*	lmo1234	-0.74246	-1.30095	-1.05527	3	3	3	3

Q8Y9N4	3-dehydroquinate dehydratase	AROD_LISMO	*aroD*	lmo0491	-0.99387	-1.05133	-1.44311	3	3	3	3
Q8Y9N5	Shikimate dehydrogenase (NADP(+))	AROE_LISMO	*aroE*	lmo0490	-1.46213	-0.75677	-0.69589	3	3	3	3
Q9S389	D-alanyl carrier protein	DLTC_LISMO	*dltC*	lmo0972	-0.83998	-3.70588	-5.20359	3	3	3	3
Q92BL2	UPF0473 protein lmo1501	Y1501_LISMO	*lmo1501*	lmo1501	-3.69463	-2.92398	-3.5011	4	4	4	4
P66219	50S ribosomal protein L33 1	RL331_LISMO	*rpmG1*	lmo1335	-4.2728	-4.76338	0.16736	4	4	4	4
Q8Y7F4	Ribosome-binding factor A	RBFA_LISMO	*rbfA*	lmo1327	-4.1022	-0.68558	-0.86976	4	4	4	4

Supplementary Table S4. Identification of DEPs of *L. monocytogenes* after H2 and H4 treatments by LC-MS/MS.

COGs Categories	Protein IDs	Name (recommend name)	Entry name (LISMO)	Gene	Locus	Fold change (log_2)	
						‹H2/C›	‹H4/C›
Amino acid transport and metabolism	Q8Y5N9	Diaminopimelate epimerase	DAPF	*dapF*	lmo2018	1.78	-
	P0DJL8	Alanine racemase	ALR	*alr*	lmo0886	-0.95*	-1.03
	Q8Y9N4	3-dehydroquinate dehydratase	AROD	*aroD*	lmo0491	-0.99*	-1.44
	Q8Y9N5	Shikimate dehydrogenase (NADP(+))	AROE	*aroE*	lmo0490	-1.46	-
	Q8Y4L2	Glycine cleavage system H protein	GCSH	*gcvH*	lmo2425	-0.82*	-1.59
Carbohydrate transport and metabolism	Q8Y4I3	Triosephosphate isomerase 1	TPIS1	*tpiA1*	lmo2457	0.72*	1.03
Cell cycle control, cell division, chromosome partitioning	Q8YAD5	Putative septation protein SpoVG 1	SP5G1	*spoVG1*	lmo0196	-	1.29
Cell wall, membrane, envelope biogenesis	Q8Y832	Ribitol-5-phosphate cytidylyltransferase	TARI	*tarI*	lmo1086	0.94*	1.29
	Q8Y5L9	UDP-N-acetylmuramoyl-L-alanyl-D-glutamate--2,6-diaminopimelate ligase	MURE	*murE*	lmo2038	-0.69*	-1.25

Coenzyme transport and metabolism	Q8Y7I9	S-ribosylhomocysteine lyase	LUXS	*luxS*	lmo1288	1.93	1.98
	Q8Y5G2	Pyridoxal 5'-phosphate synthase subunit PdxS	PDXS	*pdxS*	lmo2101	0.97*	1.15
	Q8Y6X4	Glutamyl-tRNA reductase	HEM1	*hemA*	lmo1557	-2.0	-2.11
	Q8Y564	Uroporphyrinogen decarboxylase	DCUP	*hemE*	lmo2212	-1.06	-1.20
Energy production and conversion	Q8Y4C2	ATP synthase epsilon chain	ATPE	*atpC*	lmo2528	-0.84*	-1.26
Function unknown	Q8Y3X6	Nucleoid-associated protein lmo2703	Y2703	*lmo2703*	lmo2703	1.20	2.00
	P0A4Q8	UPF0145 protein lmo0208	Y208	*lmo0208*	lmo0208	-1.56	-1.96
	Q92BL2	UPF0473 protein lmo1501	Y1501	*lmo1501*	lmo1501	-3.69	-3.50
	Q8Y6V4	UPF0173 metal-dependent hydrolase lmo1577	Y1577	*lmo1577*	lmo1577	-	-1.26
Inorganic transport and metabolism	Q8Y454	Energy-coupling factor transporter ATP-binding protein EcfA1	ECFA1	*ecfA1*	lmo2601	-2.18	-3.00
	Q8Y4L8	Methionine import ATP-binding protein MetN 2	METN2	*metN2*	lmo2419	-0.84*	-1.07
Lipid transport and metabolism	Q8Y6V9	Acetyl-coenzyme A carboxylase carboxyl	ACCA	*accA*	lmo1572	-0.85*	-1.14

		transferase subunit alpha					
Nucleotide transport and metabolism	Q8Y449	Adenylate kinase	KAD	*adk*	lmo2611	1.03	1.54
	Q8Y8N0	ATP-dependent RNA helicase CshA	CSHA	*cshA*	lmo0866	1.67	1.56
	Q8Y3T8	Probable transaldolase 1	TAL1	*tal1*	lmo2743	0.56*	2.64
	Q8Y5R1	Deoxyribose-phosphate aldolase	DEOC	*deoC*	lmo1995	-1.43	-1.54
	Q8Y6Z9	S-adenosylmethionine:tRNA ribosyltransferase-isomerase	QUEA	*queA*	lmo1531	-0.95*	-1.10
	Q8Y4A6	Homoserine kinase	KHSE	*thrB*	lmo2545	-1.24	-2.04
Post-translational modification, protein turnover, chaperone	Q9RQI6	ATP-dependent Clp protease proteolytic subunit	CLPP	*clpP*	lmo2468	1.04	1.41
	P0DJM1	Chaperone protein DnaJ	DNAJ	*dnaJ*	lmo1472	1.30	1.66
Replication, recombination and repair	Q8Y6W7	Formamidopyrimidine-DNA glycosylase	FPG	*mutM*	lmo1564	-1.15	-1.49
	Q8Y7P0	UvrABC system protein C	UVRC	*uvrC*	lmo1234	-	-1.06
Transcription	Q9RGX0	Global transcriptional regulator Spx	SPX	*spx*	lmo2191	1.02	1.73
	Q8Y673	DNA-directed RNA polymerase subunit omega	RPOZ	*rpoZ*	lmo1826	-	1.13

	P0A355	Cold shock-like protein CspLA	CSPA	*cspLA*	lmo1364	-0.70*	-1.24
	P0A357	Cold shock-like protein CspLB	CSPB	*cspLB*	lmo2016	-2.03	-3.23
	Q8Y7H7	LexA repressor	LEXA	*lexA*	lmo1302	-1.09	-1.36
	Q8Y7C6	Transcription antitermination protein NusB	NUSB	*nusB*	lmo1359	-1.15	-1.31
Translation, ribosomal structure and biogenesis	Q8Y7G7	Ribosome-recycling factor	RRF	*frr*	lmo1314	0.97*	1.14
	Q8Y7Q2	Phenylalanine--tRNA ligase alpha subunit	SYFA	*pheS*	lmo1221	1.05	0.95*
	P0DJP1	30S ribosomal protein S21	RS21	*rpsU*	lmo1469	3.41	4.52
	P0A3L1	Translation initiation factor IF-3	IF3	*infC*	lmo1785	3.75	4.08
	Q8Y443	50S ribosomal protein L24	RL24	*rplX*	lmo2621	3.36	4.08
	P66383	30S ribosomal protein S13	RS13	*rpsM*	lmo2608	2.82	3.90
	P66372	30S ribosomal protein S12	RS12	*rpsL*	lmo2656	2.18	3.47
	P66503	30S ribosomal protein S20	RS20	*rpsT*	lmo1480	2.34	3.42
	P66125	50S ribosomal protein L27	RL27	*rpmA*	lmo1540	2.98	3.31
	P66207	50S ribosomal protein L32-2	RL322	*rpmF2*	lmo2047	2.93	3.30

Q927M5	50S ribosomal protein L30	RL30	*rpmD*	lmo2614	1.97	2.88
Q8Y699	30S ribosomal protein S16	RS16	*rpsP*	lmo1797	2.24	2.82
P66611	30S ribosomal protein S7	RS7	*rpsG*	lmo2655	2.13	2.81
P66484	30S ribosomal protein S19	RS19	*rpsS*	lmo2628	2.01	2.77
Q8Y459	30S ribosomal protein S9	RS9	*rpsI*	lmo2596	2.65	2.59
Q8YAR2	50S ribosomal protein L9	RL9	*rplI*	lmo0053	2.46	2.53
Q8Y447	50S ribosomal protein L15	RL15	*rplO*	lmo2613	1.89	2.39
Q8Y444	50S ribosomal protein L6	RL6	*rplF*	lmo2617	2.02	2.22
Q8Y445	50S ribosomal protein L18	RL18	*rplR*	lmo2616	1.13	2.10
P66330	30S ribosomal protein S10	RS10	*rpsJ*	lmo2633	1.85	1.93
P66103	50S ribosomal protein L20	RL20	*rplT*	lmo1783	1.00	1.84
P60426	50S ribosomal protein L2	RL2	*rplB*	lmo2629	1.41	1.83
Q927L4	50S ribosomal protein L16	RL16	*rplP*	lmo2625	1.41	1.75
Q8Y450	50S ribosomal protein L17	RL17	*rplQ*	lmo2605	1.56	1.73
P66054	50S ribosomal protein L11	RL11	*rplK*	lmo0248	1.07	1.61
Q8Y6Y9	50S ribosomal protein L21	RL21	*rplU*	lmo1542	1.31	1.38
Q927L2	50S ribosomal protein L22	RL22	*rplV*	lmo2627	1.45	1.33
Q8Y458	50S ribosomal protein L13	RL13	*rplM*	lmo2597	1.41	1.28

P0A485	50S ribosomal protein L31 type B	RL31B	*rpmE2*	lmo2548	1.31	1.25
Q927L7	50S ribosomal protein L14	RL14	*rplN*	lmo2622	1.27	1.08
Q92C24	30S ribosomal protein S15	RS15	*rpsO*	lmo1330	1.12	1.08
P66352	30S ribosomal protein S11	RS11	*rpsK*	lmo2607	1.08	1.05
O53083	50S ribosomal protein L19	RL19	*rplS*	lmo1787	0.90*	1.02
Q8Y440	50S ribosomal protein L3	RL3	*rplC*	lmo2632	-	1.00
Q9S389	D-alanyl carrier protein	DLTC	*dltC*	lmo0972	-0.84*	-5.20
P64032	Elongation factor P	EFP	*efp*	lmo1355	-1.67	-1.96
P58817	Aspartyl/glutamyl-tRNA(Asn/Gln) amidotransferase subunit C	GATC	*gatC*	lmo1756	-2.34	-2.18
Q8Y7F4	Ribosome-binding factor A	RBFA	*rbfA*	lmo1327	-4.10	-0.87*
Q8Y6A2	Ribosome maturation factor RimM	RIMM	*rimM*	lmo1793	-0.70*	-1.09
Q8Y5S8	Ribonuclease Z	RNZ	*rnz*	lmo1977	-1.05	-
P66219	50S ribosomal protein L33 1	RL331	*rpmG1*	lmo1335	-4.27	-
Q8Y6X2	Threonine--tRNA ligase	SYT	*thrS*	lmo1559	-0.64*	-1.08

COGs: Database of Clusters of Orthologous Groups of proteins; C: control group; H2: HPP group at 200MPa; H4: HPP group at 400MPa. The number that marks "*" on the right side is $\log_2$ (fold change) value within -1~+1 and $p < 0.05$. The number that marks "-" means $p > 0.05$.

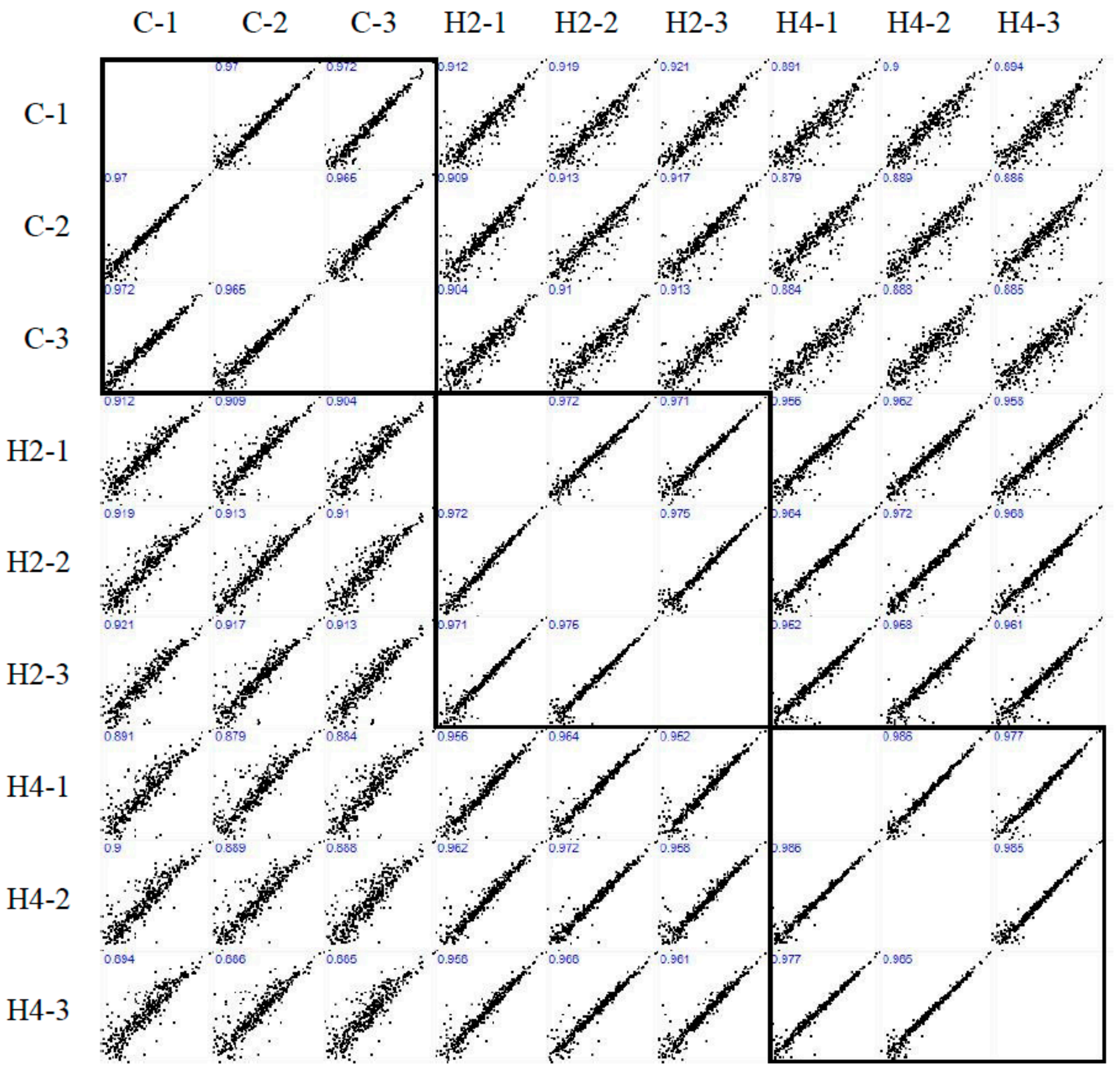

Supplementary Figure S1. The linear relationships between identified *L. monocytogenes* proteins analyzed using label-free quantitative proteomics after C, H2 and H4 treatments with triplicate. Note: C, control group; H2, HPP group (200 MPa); H4, HPP group (400 MPa). The dash after group name is the number of triplicate. Blue font is Pearson correlation coefficient. The scatter plots in the black frame is related to the triplicate of the same treatment group.

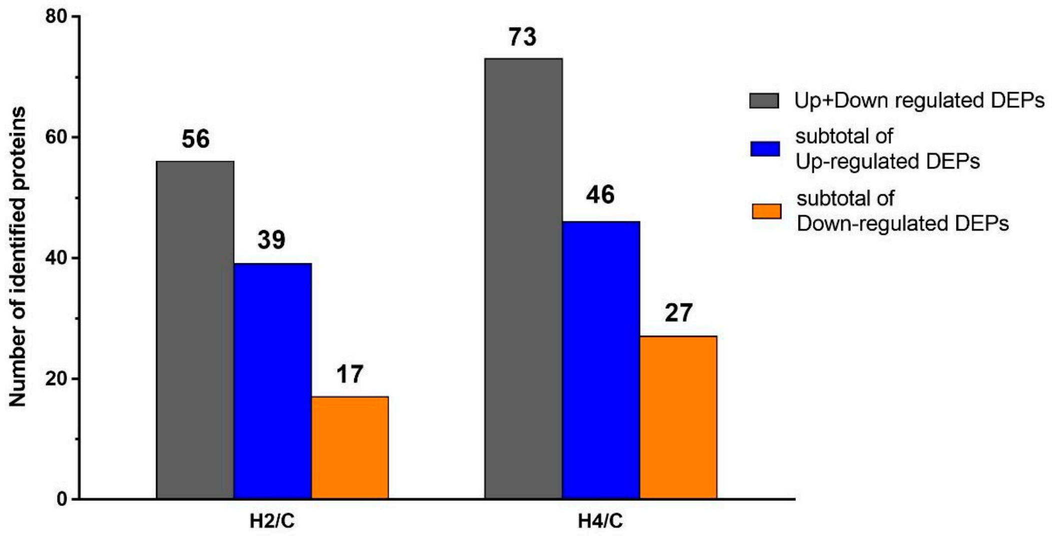

Supplementary Figure S2. The numbers of differential expressed proteins (DEPs) of *L. monocytogenes* exposed to HPP.

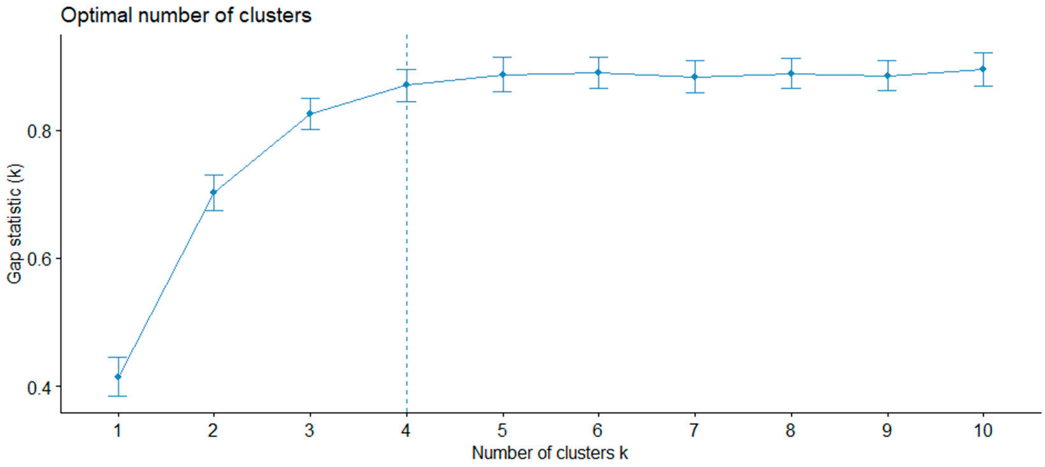

Supplementary Figure S3. The optimal number of clusters under k-means clustering algorithm.
